# Comparison of cardiac index: LiDCOrapid and PiCCOplus in the ICU

**DOI:** 10.1186/cc9482

**Published:** 2011-03-11

**Authors:** P Brass, E Mills, J Latza, J Peters, E Berendes

**Affiliations:** 1 Helios Klinikum, Krefeld, Germany; 2 LiDCO Ltd, London, UK; 3 Klinikum Duisburg, Germany

## Introduction

This study aims to compare two arterial pressure wave-form monitors: the nomogram scaled LiDCOrapid (LiDCO Ltd, London, UK) with the calibrated PiCCOPlus, (Pulsion, Munich, Germany), to determine agreement for cardiac index (CI) measurement and trending during positional changes of passive leg raise test (PLRT) and volume expansion in the SICU.

## Methods

We recruited 20 patients who had undergone major abdominal or neurosurgery and 10 patients in the SICU with progressive circulatory instability. The femoral artery was cannulated to obtain the arterial blood pressure waveform. Simultaneous measurements were made at four time points, M1 to M4: (M1) baseline, (M2) after PLRT, (M3) baseline (M4) after 500 ml Tetraspan^® ^6% over 10 minutes via pressure infusion. The PiCCO was calibrated via transpulmonary thermodilution at each time point.

## Results

Data were collected from 30 patients, age 31 to 90, ASA 2 (2), ASA 3 (24) or ASA 4 (4), BSA 1.54 to 2.52 m^2^. CI ranged from 1.5 to 7.2 l/minute/m^2 ^for PiCCO and from 1.5 to 7.1 l/min/m^2 ^for LiDCO. Regression plots were made at each time point and show good agreement across the full range of CI values (*r*^2 ^= 0.89 to 0.95). Bland-Altman analysis at each time point found low bias (10 to 50 ml/min/m^2^) and acceptable limits of agreement (16 to 30%), with the greatest difference occurring after the PLRT. Trending analysis was conducted by four-quadrant plot concordance assessment using an optimised exclusion zone of <5% ΔCI on changes at timepoints M2 to M4 relative to baseline (M1). Concordance was calculated as 97.8% overall agreement (44/45) for ΔCI >5%. Regression analysis found a high degree of correlation (*r*^2 ^= 0.86 to 0.92) and all intercepts equal to 0.

## Conclusions

In a heterogeneous patient population, LiDCOrapid CI values are in agreement with PiCCO CI values according to the accepted standard of ± 30% with minimal bias. Trending analysis showed excellent concordance of 97.8%, which meets the recently proposed standard of >90% [1]. The LiDCOrapid is a valid measure of CI and trends in CI. It is easier to set up, does not require central venous access, is independent of the arterial site and can be used both intraoperatively in the OR and in the ICU.
